# Electrochemically anodized porous silicon: Towards simple and affordable anode material for Li-ion batteries

**DOI:** 10.1038/s41598-017-08285-3

**Published:** 2017-08-11

**Authors:** T. Ikonen, T. Nissinen, E. Pohjalainen, O. Sorsa, T. Kallio, V.-P. Lehto

**Affiliations:** 1 Department of Applied Physics, University of Eastern Finland, FI-70211 Kuopio, Finland; 2 Department of Chemistry, School of Chemical Technology, Aalto University, FI-00076 Aalto, Finland

## Abstract

Silicon is being increasingly studied as the next-generation anode material for Li-ion batteries because of its ten times higher gravimetric capacity compared with the widely-used graphite. While nanoparticles and other nanostructured silicon materials often exhibit good cyclability, their volumetric capacity tends to be worse or similar than that of graphite. Furthermore, these materials are commonly complicated and expensive to produce. An effortless way to produce nanostructured silicon is electrochemical anodization. However, there is no systematic study how various material properties affect its performance in LIBs. In the present study, the effects of particle size, surface passivation and boron doping degree were evaluated for the mesoporous silicon with relatively low porosity of 50%. This porosity value was estimated to be the lowest value for the silicon material that still can accommodate the substantial volume change during the charge/discharge cycling. The optimal particle size was between 10–20 µm, the carbide layer enhanced the rate capability by improving the lithiation kinetics, and higher levels of boron doping were beneficial for obtaining higher specific capacity at lower rates. Comparison of pristine and cycled electrodes revealed the loss of electrical contact and electrolyte decay to be the major contributors to the capacity decay.

## Introduction

Lithium ion batteries are widely used in mobile electronics and are considered to be one of the best choices as a power source for electric vehicles because of their high energy density. Secondary batteries may also become one of the key factors in controlling climate change as they can store energy produced by intermittent renewable sources^1, 2^. An optimal battery should have several key characteristics: low price, safety, high energy density, fast charging and discharging capability, and good durability^3^.

One promising approach to improve the capacity of lithium-ion batteries is to use silicon (Si) instead of the more traditional graphite as the anode material. Silicon has a theoretical specific capacity of ~4200 mAh/g for lithium whereas the corresponding value for carbon is only ~370 mAh/g. Even when taking into account the volumetric expansion, simply by changing the graphitic anode to its silicon version, it has been estimated that the cell energy density could be improved by 10–30%^4^. Accordingly, good results regarding capacity retention and rate capability have been reported^5, 6^. Unfortunately, generally these results have been achieved by creating complex compositions (e.g. core-shell structures), which often have insufficient volumetric capacities^7^. The low volumetric capacities arise from the fact that most of the studied anodes contain very low loadings of highly porous silicon. These anode materials typically show good capacity retention for even thousands of cycles but the main reason for their good performance is due to the low mass loading of the active material, mitigating large dimensional changes upon potential cycling. There is an ever increasing need to devise more practical anode materials especially with respect to their volumetric and areal capacities^8, 9^. The unresolved problem associated with novel, high theoretical capacity materials, such as silicon, is whether one can achieve good capacity retention with a high mass loading of the active material while keeping also the volumetric capacity high. To address this issue, systematic studies are needed to clarify the underlying limitations in the silicon anode structures.

One way to acquire the aforementioned characteristics is to use silicon microparticles containing nano-sized pores – mesoporous silicon (PSi)^9^. It is recognized that porosity should be as low as possible if one wishes to maximize volumetric capacity but nonetheless high enough to accommodate the volume change during charging/discharging. The present study investigated the effects of microparticle sizes of PSi and chemical surface modifications on the battery performance. While it is commonly accepted that solid Si nanoparticles will perform better as an anode material than solid Si microparticles, as far as we are aware, there are no systematic studies on effect of Si particles’ porosity on their behavior. Contrary to general expectations, we found that intermediately sized microparticles performed significantly better than smaller particles close to the sub-micron range. Furthermore, we discovered that thermally carbonized surface of porous silicon enhances the lithiation kinetics of the material at the rate of 2 A/g.

## Results and Discussion

### Porous silicon design and electrodes

By designing the porous structure of the silicon particles to withstand the volumetric change, it may be possible to achieve the benefits associated with micrometer scale mesoporous silicon particles in Li-ion battery anodes so that these can replace the traditional graphite anodes in a variety of applications. The preparation of PSi by electrochemical etching was chosen here because of its good repeatability and its ability to produce pores with well-defined characteristics. The aimed porosity (the volume of the parallel pores compared with the volume of the particle) of the particles was 50% as theoretically this should accommodate the 300% volume change of the particle due to lithiation. Furthermore, in this way the volumetric capacity could be maximized while minimizing the reduction in the mechanical properties of the particles. However, in the direction parallel to the pores, the thickness of the particle should vary by 44% (cf. Supplementary Information). The method is a good way to produce different porous structures for screening purposes, although it is obvious that the final PSi product with a predefined structure should be manufactured with cheaper methods^10, 11^.

Shortly, PSi particles were prepared by electrochemical etching and subsequent planetary ball milling (Fig. S1, Supplementary Information). All PSi samples in the present work had similar pores and porosities unless otherwise mentioned (Table S1, Supplementary Information). The porosity of each sample was around 50% while the aveage pore size ranged between 5–16 nm. Surface modifications were conducted in a tube oven under a nitrogen atmosphere. The electrodes were prepared by the slurry casting technique and 2016 coin cells were used in the electrochemical measurements (Supplementary Information).

Three different surface modifications were produced to investigate their effects on the performance of the electrodes: As-anodized porous silicon (AAPSi), thermally hydrocarbonized porous silicon (THCPSi) and thermally carbonized porous silicon (TCPSi). AAPSi represented a sample of hydrogen-terminated surface obtained directly after electrochemical etching. AAPSi is expected to be oxidized to some extent in ambient air. The THCPSi sample has a hydrocarbon layer covering the silicon framework. The TCPSi sample represents one step further in terms of stability; this has a thin silicon carbide/silicon oxycarbide layer covering the surface of the PSi particles. The outermost layer of TCPSi consists of disordered graphitic carbon^12^.

**Figure 1:**
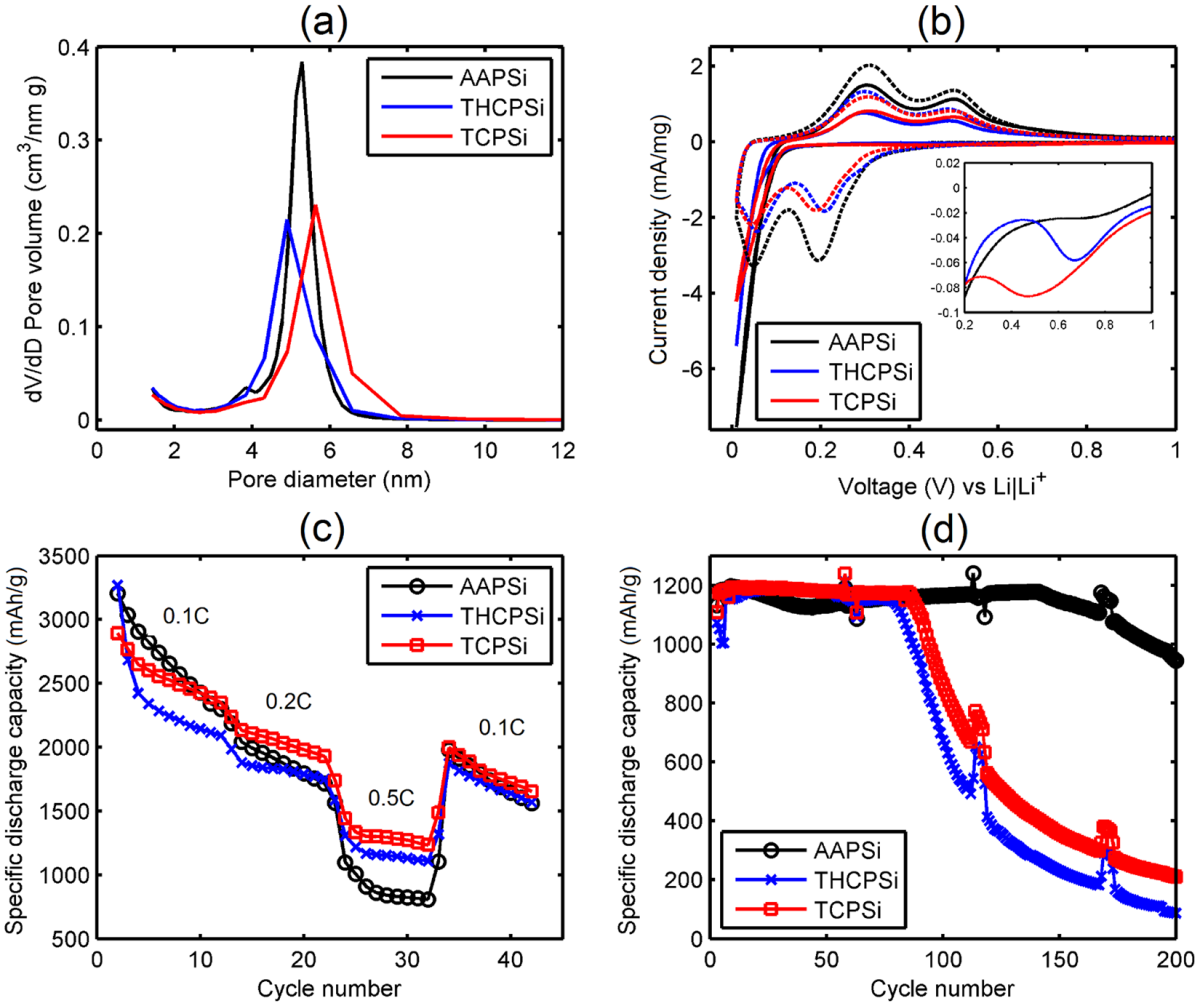
PSi pore size distribution and electrochemical measurements for different surface modifications. (**a**) Pore size distributions of the AAPSi, THCPSi and TCPSi samples. (**b**) Cyclic voltammetry results from scan #1 (solid line) and scan #4 (dashed line). Inset shows formation of SEI layer during the first scan. (**c**) Galvanostatic rate capability results for the AAPSi, THCPSi and TCPSi samples. Mass loading of silicon is 1.0 mg/cm^2^. (**d**) Galvanostatic capacity retention results with limited capacity of 1200 mAh/g. Cycling was done periodically: 5 × 0.1 C + 50 × 0.2 C while 1 C equals to 4200 mA/g. Mass loading of silicon is 0.6 mg/cm^2^.

According to the pore size distributions (Fig. 1a), the pore diameters were approx. 5–6 nm. The porosity of these samples was approx. 50% as intended. While a higher porosity might enhance the cyclability of the material, it would also lower its volumetric capacity, which ultimately determines the usability of the material^4^. Pore size was in the mesoporous range which is large enough for the solvated lithium ions to pass through the pores easily^13^. The tap densities of the PSi samples were ca. 0.68 g/cm^3^ regardless of the surface modification (Table S2, Supplementary Information). As the bulk density of silicon is 2.33 g/cm^3^, the measured tap density was quite high for a porous material with relatively large particles. In literature, the tap densities of the nanostructured silicon used in Li-ion battery anodes are typically much lower^14, 15^.

### Electrochemical results

Figure 1b shows the cyclic voltammetry results. During the first scan, all the samples are lithiated below 0.1 V as depicted by a strong cathodic peak close to 0.01 V. In the following cycles, two cathodic peaks can be seen at 0.05 V and at 0.2 V indicating a two-step reaction after crystalline silicon has become amorphous (at least partially) during the first scan. The anodic peaks at 0.3 V and 0.5 V correspond to delithiation of Li_x_Si compounds. Interesting phenomena related to solid electrolyte interface (SEI) formation are shown in the inset of Fig. 1b.

During the first scan, a SEI formation peak could be detected for THCPSi at around 0.7 V while for TCPSi, a SEI formation peak was observed at 0.5 V. For the AAPSi sample, no clear SEI formation was present leading to the conclusion that the SEI layer formation had been induced by surface carbon. The half-peak potentials for carbonized samples were on average 12 mV higher for cathodic peaks whereas for anodic peaks, the half-wave potentials were on average 2 mV lower than seen with the AAPSi sample. This suggests enhanced kinetics for THCPSi and TCPSi samples, especially during the lithiation step. While the kinetics of AAPSi sample was inferior, it exhibited much higher peak values, reflecting its higher capacity and reactivity. This was confirmed by calculating the capacity based on the area under the anodic peaks. After full activation (scan #4), the anodic capacity for AAPSi was 3500 mAh/g while for THCPSi and TCPSi it was only 2400 mAh/g and 1900 mAh/g, respectively. The capacities for each scan can be seen in Table S3 (Supplementary Information). These results indicate that thermal carbonization treatment also deactivates parts of the material available for lithiation in the CV measurement.

Galvanostatic cycling results determined in the rate capability experiments (Fig. 1c) support the findings based on CV. At higher charging rates, THCPSi and TCPSi samples show higher capacity than AAPSi with this being most clearly seen at a current density of 2100 mA/g where the difference in capacity was over 400 mAh/g between TCPSi and AAPSi. A limited capacity cycling at lower charging rates was used to evaluate capacity retention (Fig. 1d) and a profound difference was detected between the different surface modifications. AAPSi outperformed THCPSi and TCPSi samples by supplying a stable capacity of 1200 mAh/g for more than 140 cycles while the other two samples could only supply a stable capacity for fewer than 90 cycles.

**Figure 2:**
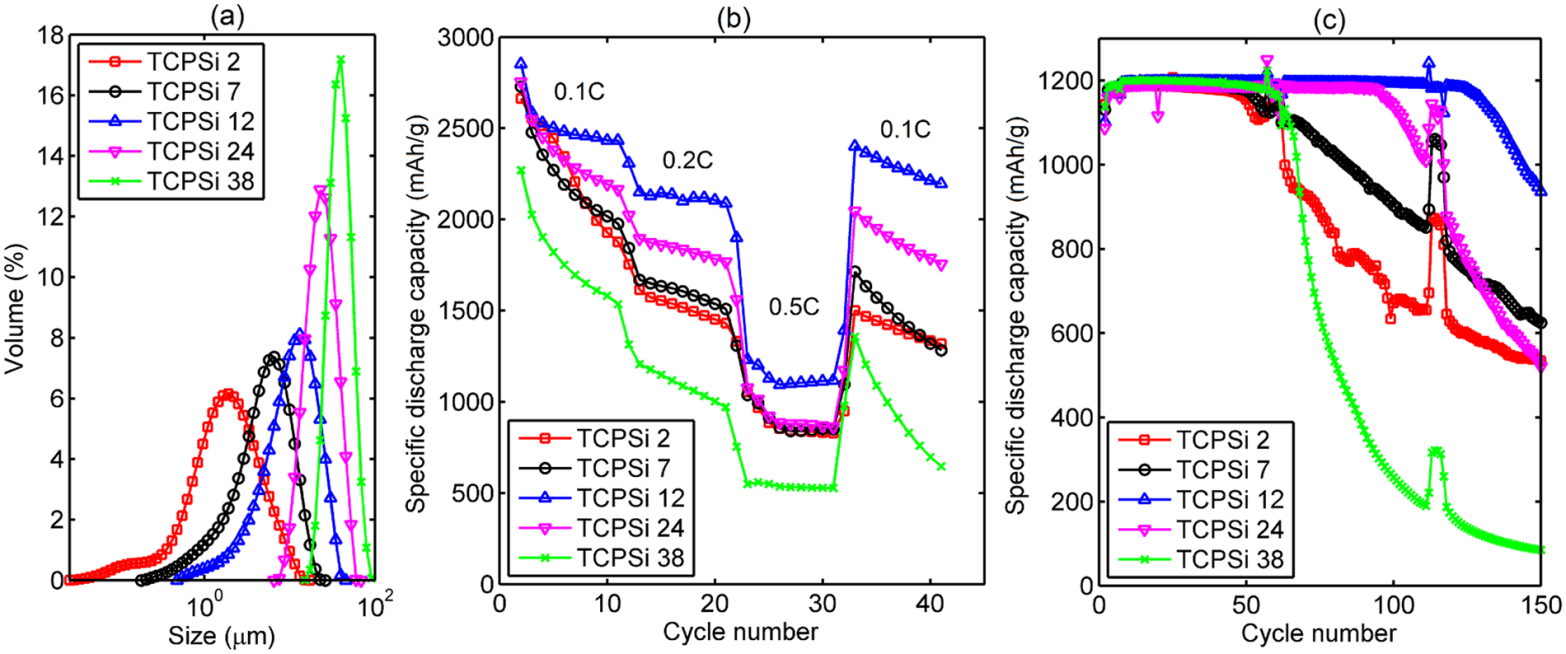
PSi particle size distributions and corresponding electrochemical cycling results for different sizes. (**a**) Particle size distributions for the TCPSi samples. (**b**) Galvanostatic rate capability results for the TCPSi samples. Mass loading of silicon was 1.1 mg/cm^2^. (**c**) Galvanostatic capacity results with a limited capacity of 1200 mAh/g. Cycling was done periodically: 5 × 0.1 C + 50 × 0.2 C while 1 C equals to 4200 mA/g. Mass loading of silicon was 0.8 mg/cm^2^.

To study the effect of particle size on the performance, TCPSi samples were chosen because of their enhanced kinetics based on the earlier findings in both CV and galvanostatic cycling when compared with AAPSi (Fig. 1b–c). Five distinct size distributions were evaluated (Fig. 2a).

Based on galvanostatic cycling (Fig. 2b–c), the best performing particle size was determined to be 12 µm. A decrease in the particle size exerted a stronger effect than its increase as indicated by the lower rate performance (Fig. 2b) and faster capacity fade in the longer cycling (Fig. 2c). TCPSi 12 was able to recover the capacity after higher rate cycling (Fig. 2b) indicating that the silicon material had not broken apart during the prior cycles. It seems that a particle size of 12 µm achieves an optimal external surface area (0.9 m^2^/g, Table S4 (Supplementary Information)) in relation to the binders of CMC and PAA utilized in the electrodes. It has been reported recently that the interaction between the particles and the binder materials is critical regarding the capacity retention^16^. Another thing to consider is the thickness of the electrode. In the present study, the thickness of active material layer on copper foil varied from 20 to 30 µm. Thus, it is reasonable that the particles with the diameter corresponding the thickness of the electrode would not perform that well. On the other hand, the particles with much smaller diameter will need an effective galvanic connection to the current collector. Capacity retention results for the AAPSi and THCPSi samples reveal similar behavior in relation to the size of the PSi particles (Fig. S2, Supplementary Information).

In addition, the effects of different doping degree and enlargement of the pores were studied with THCPSi + 12 and ANTCPSi + 12 samples (20 mΩ cm, higher resistivity). Firstly, lowering the boron dopant in the wafers resulted in poorer performance at lower charging rates (Fig. S3, Supplementary Information) due to the lower conductivity of the material. Secondly, even though annealing enlarged the pores of PSi, the cycling performance was less impressive as indicated by the rapidly deteriorating capacity (Fig. S3, Supplementary Information). This clearly shows that larger pores produced by annealing are not beneficial for battery applications as annealing also results in a thickening of the pore walls. It should be noted that annealing of PSi also makes the material more brittle which also contributes to the rapid fading in the capacity.

### Post-characterization

In the X-ray diffraction patterns, three peaks could be detected; these corresponded to the cubic silicon crystalline structure (2θ = 28.4°, 47.3° and 56.1°; Fig. S4a, Supplememtary Information). Figure S4b shows the diffractograms from cycled and delithiated electrodes after 42 cycles. The peaks originating from silicon crystalline structure can no longer be detected as the crystalline silicon had been converted into an amorphous phase during cycling. Two small peaks originating from lithium carbonate (Li_2_CO_3_) were observed at 2θ angles of 30.5° and 31.8° for the AAPSi 2 sample (Fig. S4c). The TCPSi 2 sample, however, did not display any peaks other than those related to the current collector (copper foil). This indicates that Li_2_CO_3_ had been formed during the lithiation process on the electrodes without the protective carbon layer.

Both fresh and cycled (delithiated) electrode materials were characterized with FTIR to examine whether there were any other reasons, such as Li-ion entrapment or SEI-decomposition, for the capacity fade of the anode. The results obtained are shown in Fig. S5 (Supplementary Information). The unused electrode material displayed peaks arising from carboxylic group vibrations (-COOH) at 1700 cm^−1^ and a peak at 3430 cm^−1^ due to the –OH group. Carboxylic groups could be found in both PAA and CMC which are used as binder materials. The peak at 1040 cm^−1^ is a characteristic of silicon-oxygen bonding (Si-O-Si)^17^. In the cycled electrode material, a strong presence of Li_2_CO_3_ was observed as indicated by the peaks around 860 cm^−1^, 1430 cm^−1^ and 1490 cm^−1^ ^18^.

Based on XRD, only lithiated electrodes exhibited clear diffraction peaks characteristic to Li_2_CO_3_ and the formation of crystalline Li_2_CO_3_ had occurred during the lithiation of AAPSi. It is worth noting that Li_2_CO_3_ is also formed when Li is exposed to ambient air^19^. However, traces of Li_2_CO_3_ have also been found in photoelectron spectroscopy without exposure to air^20^. There were other significant peaks present in the spectra of the cycled electrode material at 1630–1640 cm^−1^ and at 1330 cm^−1^ indicating the presence of RCOOLi^18^. The latter peak was hard to distinguish because of the shoulder related to the strong peak of Li_2_CO_3_ at 1430 cm^−1^. Both RCOOLi and Li_2_CO_3_ are well known reduction products of EC and DMC^18, 21^. Thus it is most likely that the unstable SEI also contributed to the capacity fade of the PSi based electrodes which is reflected in the continuous fade in all of the electrochemical measurements (Figs 1–2). These results support the earlier findings which proposed a similar decomposition mechanism^22, 23^. In addition to electrolyte decomposition, gas formation (CO_2_, H_2_) inside the cells due to exchange of ions can also cause a capacity decay since the coin cells used here did not have any relief valve. H_2_ formation is suspected for PAA with ion exchange (PAA can release H^+^) and CO_2_ can be formed when lithium is oxidized to Li_2_CO_3_^19^.

### Particle size and morphology evaluation

**Figure 3:**
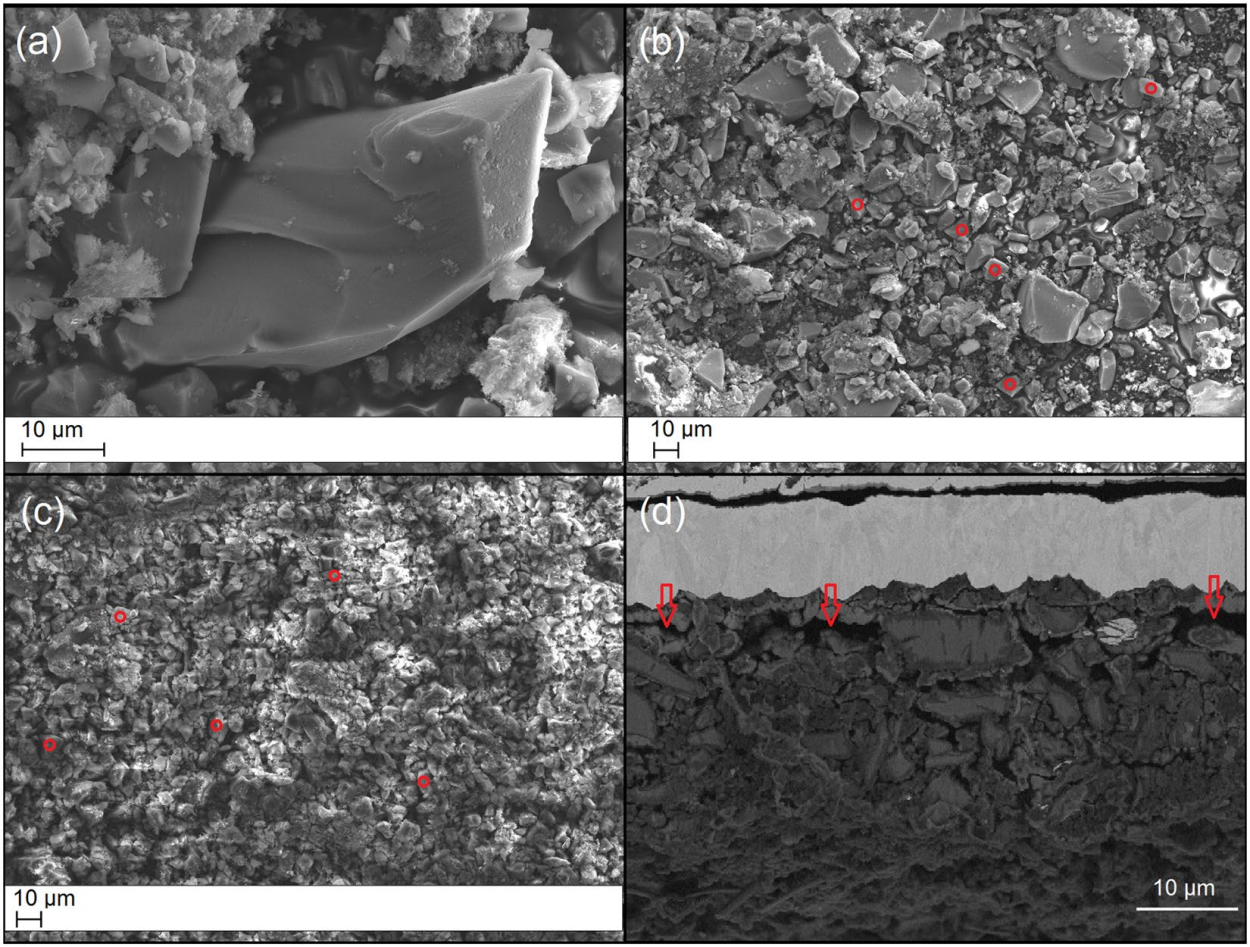
SEM images of non-cycled and cycled particles/electrode. SEM images of non-cycled particles (**a**–**b**) and particles cycled for 42 cycles (**c**). After cycling, the edges of the particles become uneven and somewhat blurry. A few 10 µm sized particles are indicated by red circles. Other objects in the images are from CB or from binder materials (CMC and PAA). (**d**) Side view from an ion cut electrode from TCPSi 38 sample after 300 cycles. The top part is the copper current collector. Empty spaces between the current collector and active material are indicated by red arrows.

To investigate the morphology of the PSi particles after cycling, SEM was used on the particles gathered from the TCPSi 38 sample electrodes (Fig. 3). The comparison of the particle sizes between different cycles revealed a tendency for the largest particles to break down while the mode of particle size in the images (around 10 µm) remained unchanged after 42 cycles (Fig. 3a–c). This supports the results regarding optimal particle size for PSi. A delithiated TCPSi 38 electrode from the longer cycling experiment was cut with ion bombardment and the resulting side view from the cycled electrode after 300 cycles is shown in Fig. 3d. The electrode has considerable amounts of empty space between the active material and the current collector leading to loss of contact between them.

**Figure 4:**
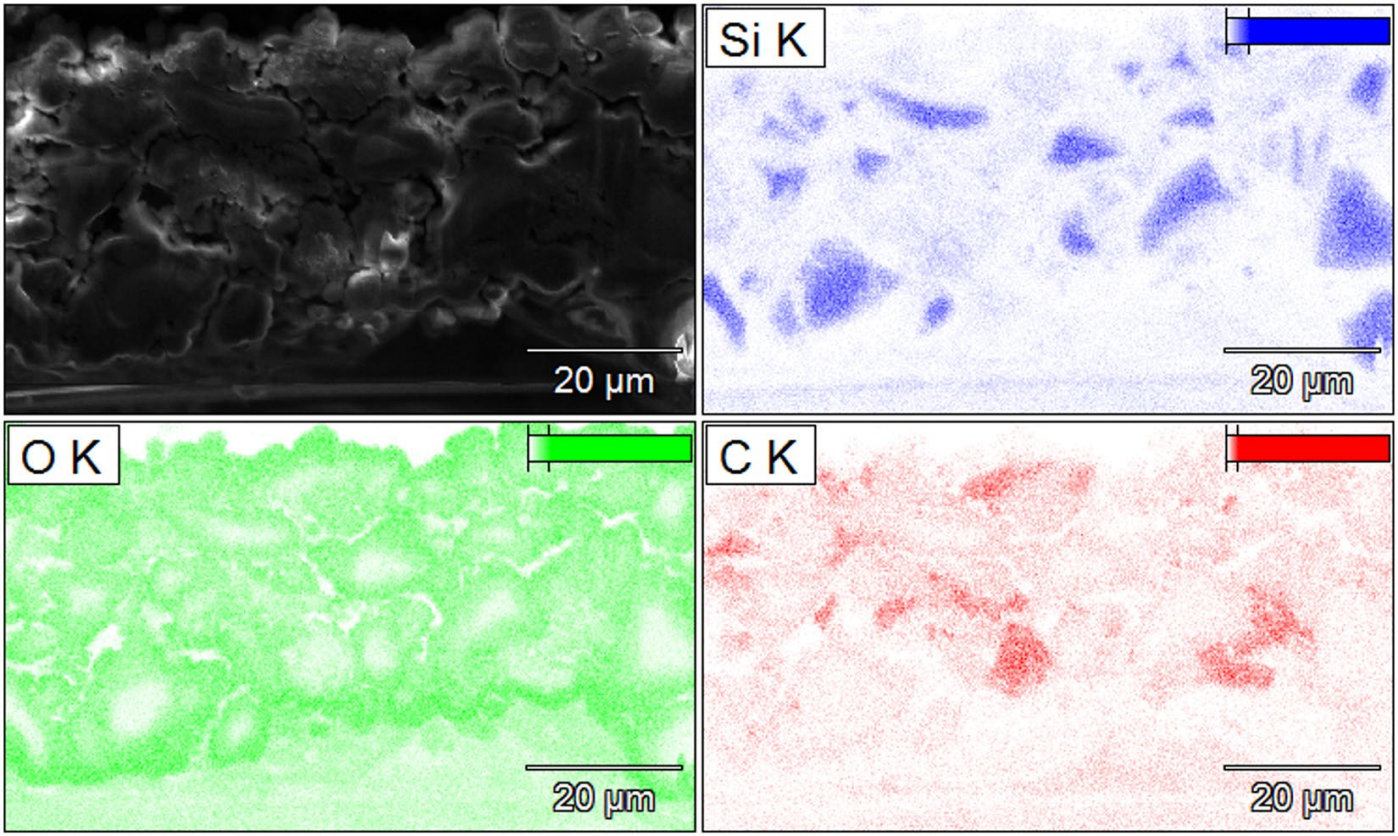
SEM image and corresponding EDS mapping from ion cut TCPSi electrode after 300 cycles. Silicon (Si), oxygen (O) and carbon (C) mapping after 300 cycles.

TCPSi and TCPSi 38 samples were then investigated with EDS to analyze the mechanism behind capacity decay. Based on the results (Figs 4 and S6), intact PSi particles are still present in the anode even after 300 cycles. TCPSi microparticles can at least to some extent withstand the volumetric changes during lithiation and delithiation. Based on the ion cut TCPSi sample, the mean particle diameter is around 8 µm while the original mean diameter in the sample is 13 µm. Additionally, carbon from binder materials and conductive additive could be seen as clusters in the cycled samples when compared to the fresh ion cut THCPSi (Fig. S7, Supplementary Information). Despite the rapid capacity decay of the sample, individual silicon particles could still be identified from the cycled electrode. This indicates that silicon particle cracking was not the only reason for the capacity decay even in the worst performing sample. Supposedly, the loss of electronic contact due to cycling inhibits the electrode from functioning properly causing PSi material to be insulated from the current collector.

**Figure 5:**
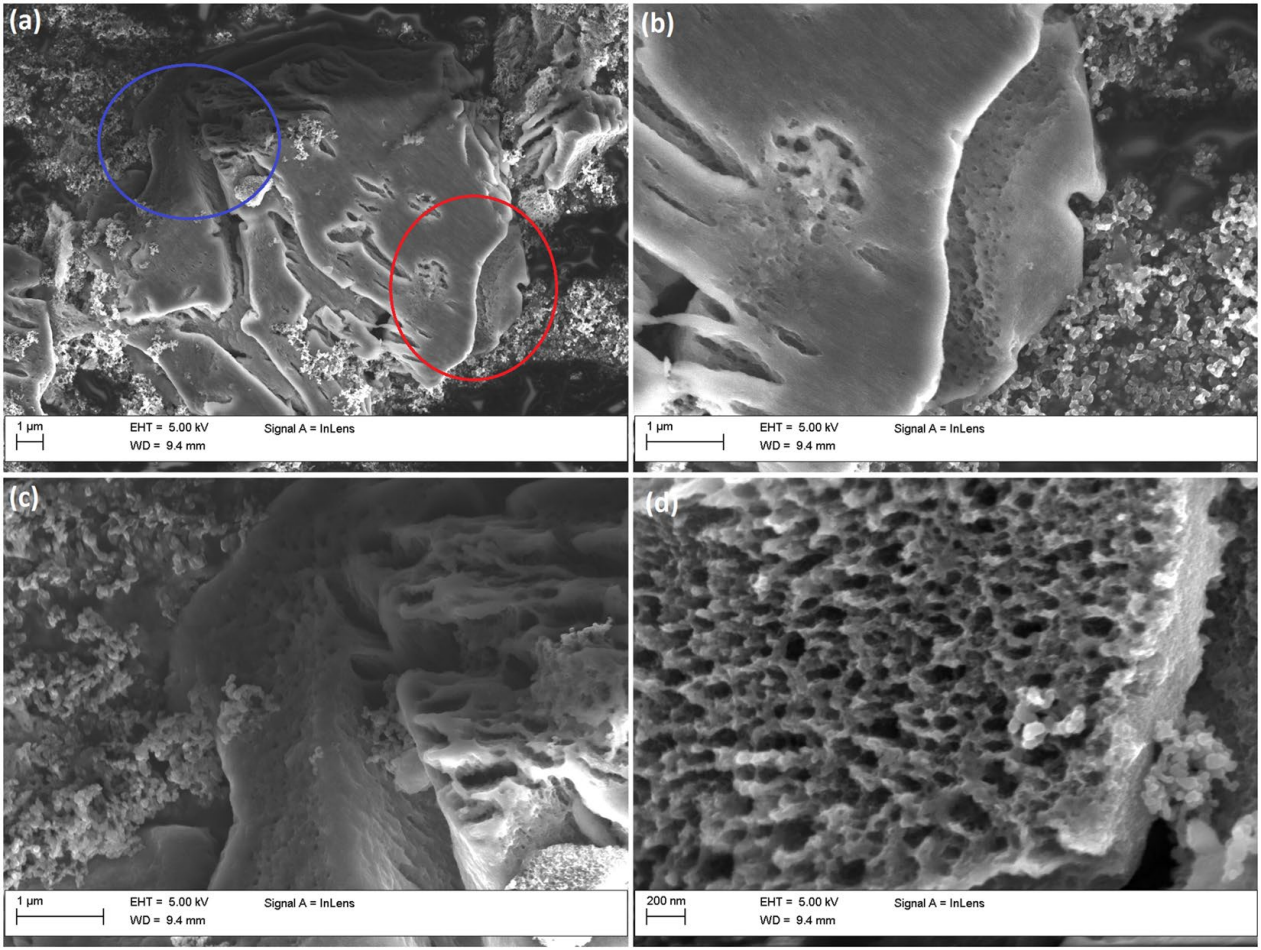
SEM images of particles after 300 cycles. SEM images from individual particles after 300 cycles from TCPSi 38 sample. (**a**) Cracks can be seen in the PSi particle. (**b**) A larger magnification image from (**a**) indicated by the red circle. Pore openings can be seen under the vertical crack in the middle of the image. (**c**) A larger magnification image from (**a**) indicated by a blue circle. Pore openings can be seen on the cracked particle in the middle. (**d**) Top-down view into a cycled particle with enlarged pore openings of approximately 50 nm in diameter.

To explore the degradation mechanism of the PSi particles, individual particles were chosen for a more detailed SEM study from sample TCPSi 38. Based on the cracks seen on PSi (Fig. 5a and c) it seemed evident that they tended to form in a non-parallel direction to the pores. After 300 cycles the pore openings of the particles were also significantly larger than in the case of the original particles; this can be clearly seen in the SEM images with average pore opening of 50 nm in diameter (Fig. 5b and d), a tenfold increase in comparison to the original pore size of the material.

## Conclusions

The optimal size for porous particles was between 10 µm and 20 µm based on the galvanostatic cycling. CV and galvanostatic cycling clearly indicated that the presence of a silicon carbide layer enhanced the kinetics of lithiation which is beneficial for fast charging/discharging. A lower degree of boron doping of silicon was disadvantageous for battery application. Pore enlargement through annealing dramatically deteriorated the performance of PSi. Cracking of PSi particles, the loss of electrical contact especially between the current collector and the anode itself, and the instability of the SEI layer were found to be the main reasons for capacity fade during cycling based on the FTIR, XRD and SEM/EDS studies.

## Methods

### Porous silicon preparation and surface modifications

PSi was prepared by electrochemical etching of boron-doped silicon wafers with <100> crystal orientation and wafer resistivity of 3 mΩ cm (Okmetic). The etching was realized by a constant current density in an aqueous 38% hydrofluoric acid (VWR)/ethanol (Altia) electrolyte (3:1, v-v). A less doped silicon wafer (20 mΩ cm, Okmetic) was used to produce two other samples to evaluate the effect of doping degree and enlargening of the pores. The separation of the porous layer from the substrate was achieved with high current pulses of 160 and 250 mA/cm² ^24^. Porous films were comminuted first in a mortar and further milled with a planetary ball mill (Fritsch Pulverisette 7). The zirconium oxide milling balls utilized here were 10 mm in diameter. The particles were sieved through 25 µm, 10 µm and/or 4 µm sieves (Precision Eforming) depending on the final size of the prepared batch. Sieving was performed both before and after surface passivation of the particles to avoid agglomeration.

The surface modification of the particles was made after dipping the particles in hydrofluoric acid (HF) to remove the oxide layer from PSi. Annealing (AN), thermal hydrocarbonization (THC) and thermal carbonization (TC) were performed under nitrogen atmosphere. To anneal the sample, PSi was flushed with nitrogen for 30 minutes in room temperature in a quartz tube. The particles were put inside a tube oven at 600 °C for 45 minutes. The sample was afterwards cooled to room temperature. Thermal hydrocarbonization was started with similar 30 minutes flushing of nitrogen as with the annealing treatment. Acetylene flow was then added while keeping the nitrogen flow open. After 15 minutes the sample was inserted into tube oven at 500 °C and left there for 14.5 minutes. The acetylene flow was subsequently switched off, and after 30 seconds the sample was cooled down in room temperature. Thermal carbonization was made after this when needed. Acetylene flow (and nitrogen flow) was opened for 9 minutes and 40 seconds. For the last 20 seconds the sample was flushed only with nitrogen. Finally, the sample was located inside the tube oven at 820 °C for 10 minutes and then cooled down to room temperature.

### Electrode preparation and electrochemical measurements

Electrodes were prepared by mixing PSi particles, carboxymethyl cellulose (CMC, Sigma Aldrich), polyacrylic acid binders (PAA, Sigma Aldrich) and conductive carbon black (CB, C65, Timcal) in deionized water. The ratio applied was 60 w-% of PSi, 10 w-% of CMC, 10 w-% of PAA and 20 w-% of CB. The method is similar as described by Cho *et al*.^25^. The prepared slurry was then spread onto a copper foil (14 µm thick) using a Doctorblade coating machine for wet thicknesses of 120 µm and 80 µm. The prepared electrode sheet was subsequently dried for two hours at 150 °C in vacuum. Thereafter the electrodes were cut with an electrode punching tool (El-Cell). Next, the electrodes were placed in a vacuum oven at 110 °C and left there overnight. After cooling the electrodes, they were moved in the vacuum chamber to an argon glove box. The silicon loading in the electrodes was either 1.0–1.1 mg/cm^2^ for rate capability measurements or 0.6–0.8 mg/cm2 for limited capacity cycling. The electrode thickness without copper foil was between 20–30 µm depending on the mass loading. The 2016 type coin cells were assembled in the glove box. The electrolyte used was 1 M LiPF_6_ in 1:1 ethylene carbonate (EC): dimethyl carbonate (DMC) (LP30, Merck). A fiberglass sheet (GF/A, Whatman) with a thickness of 260 µm served as a separator. Counter and reference electrode was of lithium metal (0.38 mm in thickness, Sigma-Aldrich).

Cyclic voltammetry (CV) was measured with Autolab PGSTA302N. Each sample was measured for 10 cycles between 0.01 and 2 V with 0.05 mV/s scan rate. Galvanostatic experiments were performed with a Neware battery testing device at room temperature. Charging and discharging speed was controlled using the theoretical capacity of silicon (4200 mAh/g). Only the capacity of silicon was taken into account in calculations. The electrochemical experiments were done between 0.01 and 2 V. With longer cycling experiments the capacity was limited to 1200 mAh/g. After cycling, the cells were opened in the glove box and the anodes were washed three times with DMC to flush away the electrolyte for further materials characterization.

### Characterization

PSi particles were characterized with gas adsorption measurements (Micromeritics Tristar II 3020) to obtain pore size distributions and particle sizes were evaluated with Mastersizer 2000 with Hydro 2000S accessory for wet sample dispersion. Tap density was measured by placing a defined mass of sample powder in a graduated cylinder and tapped until the volume of sample powder did not change anymore. FTIR experiments were carried with transmission setup utilizing KBr tablets made from electrode material. The instrument used was Thermo Nicolet Nexus 8700 and the resolution was 2 cm^−1^. For XRD measurements Bruker D8 Discover was used with copper tube and the measurement setup was grazing incidence diffraction (GID) with Göbel mirror with 8° angle of incidence. SEM imaging was done using SE and InLens detectors with 12 kV and 5 kV acceleration voltage, respectively. The instrument was Zeiss Sigma HD VP. The PSi particle samples imaged with SEM were prepared from electrodes by scraping the delithiated active material from the copper foil. The material was then dispersed in ethanol, and after a few minutes, the supernatant was taken out and the sediment was dried. The same protocol was repeated for the second time for the sediment with diluted HCl (Alfa Aesar) to remove the oxide layer from the particles. The ion cut sample was prepared with Leica EM RES102 ion beam milling system.

### Data availability

Data available from V.-P. L. (vesa-pekka.lehto@uef.fi).

## Electronic supplementary material

Supplementary information
