# Supplementary material for: Electrochemically anodized porous silicon: Towards simple and affordable anode material for Li-ion batteries
Source: Sci Rep. 2017 Aug 11;7:7880. doi: 10.1038/s41598-017-08285-3 (PMC5554169; doi:10.1038/s41598-017-08285-3)
Supplement: Supplementary file 1 — Supplementary information [file 41598_2017_8285_MOESM1_ESM.pdf]

Supplementary Information

Electrochemically anodized porous silicon: Towards simple and affordable anode material for Li-ion batteries

T. Ikonen, T. Nissinen, E. Pohjalainen, O. Sorsa, T. Kallio and V.-P. Lehto

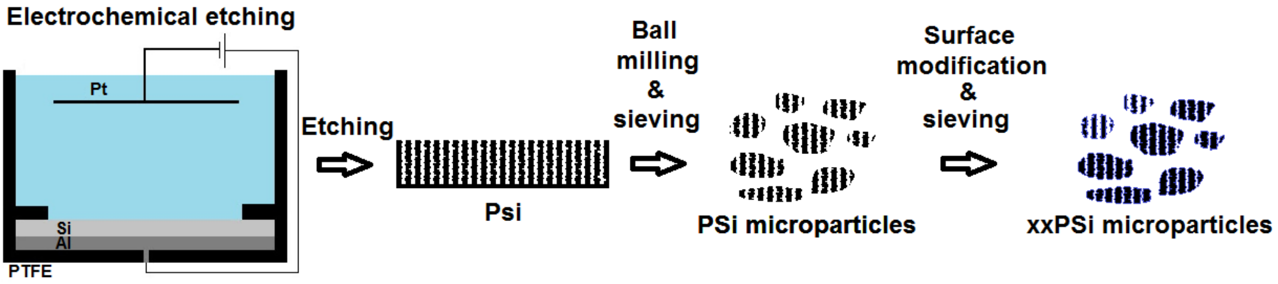

Figure S1. Schematic for the preparation and surface modification of PSi microparticles used as the Li-ion anode material.

Calculation of optimal porosity for pore structure

Consider a 3D porous network where cylindrical pores are parallel to each other along *z* direction. Assuming the volumetric expansion of silicon to be 300 % with uniform volume expansion in all three directions, the expansion in each orthogonal direction is $\sqrt[3]{3}$. Since the pores can only take the volume expansion in the *x* and *y* directions, the expansion in 2D is $\left(\sqrt[3]{3}\right)^2$. The porosity needed to absorb this expansion is then $1 - \left(1/\left(\sqrt[3]{3}\right)^2\right) \approx 0.52$. Thus 52 % porosity will counter the volume expansion in two of the three orthogonal directions leaving only the *z* direction expansion to be approximately 44 %.

Table S1. Samples used in the study.

Sample	Wafer resistivity (mΩ cm)	PSi surface	Porosity (%)	Average pore diameter (nm)	BET surface area (m^2/g)	Volume weighted mean particle diameter (µm)*
**AAPSi**	3	AA	51	6	193	19
**THCPSi**	3	THC	45	5	196	13
**TCPSi**	3	TC	48	6	209	13
**AAPSi 2**	3	AA	41	7	123	2
**THCPSi 3**	3	THC	54	9	220	3
**TCPSi 2**	3	TC	52	9	205	2
**AAPSi 6**	3	AA	47	6	179	6
**THCPSi 6**	3	THC	47	6	196	6
**TCPSi 7**	3	TC	46	6	195	7
**TCPSi 12**	3	TC	41	5	204	12
**ANTCPSi+ 12**	20	TC	57	16	124	12
**THCPSi+ 12**	20	THC	51	8	219	12
**THCPSi 12**	3	THC	41	5	205	12
**TCPSi 24**	3	TC	43	5	192	24
**TCPSi 38**	3	TC	42	5	197	38

* Volume weighted mean particle diameter is calculated by

$$D[4,3] = \frac{\sum_1^n D_i^4 n_i}{\sum_1^n D_i^3 n_i}$$

where D_i is diameter of a particle and n_i is the number of particles with that specific diameter.

Tap density study

Tap density was measured by placing a defined mass of sample powder in a graduated cylinder and tapped until the volume of sample powder did not change anymore. Bulk density was measured before tapping.

Table S2. Tap density results with mean and standard deviation (n = 3).

Sample	Bulk density (g/cm^3)	Tap density (g/cm^3)	Density based on gas adsorption measurement (g/cm^3)*	BET surface area (m^2/g)
**AAPSi**	0.49 ± 0.06	0.68 ± 0.02	1.15 ± 0.02	196 ± 3
**THCPSi**	0.54 ± 0.003	0.67 ± 0.02	1.21 ± 0.02	192 ± 1
**TCPSi**	0.52 ± 0.02	0.68 ± 0.03	1.19 ± 0.03	204 ± 1

* After measuring specific volume of pores (v_P, cm^3/g) inside PSi with gas adsorption, density of PSi material can be calculated by:

$$\rho_{PSi} = 1/(v_P + 1/\rho_{Si})$$

where ρ_{Si} is density of Si (2.33 g/cm^3).

Calculation of silicon content in PSi material

The elemental silicon content of the thermally carbonized samples (TCPSi) was evaluated based on density of silicon carbide (SiC), silicon (Si) and TCPSi. We assumed that the pore walls have a SiC layer of thickness *d* and the rest of the material is of pure Si. As the densities of both SiC and Si are known from literature, and the density of TCPSi is already reported[1], the elemental Si content of the TCPSi samples was thus calculated with:

$$w\%_{Si} = (\rho_{SiC} - \rho_{TCPSi})/(\rho_{SiC} - \rho_{Si})$$

With the densities of 3.21 g/cm^3, 2.44 g/cm^3 and 2.33 g/cm^3 for SiC, TCPSi and Si, respectively, the elemental silicon content of TCPSi was estimated to be ca. 90 w-%. The same value was assumed to be valid for all different samples utilized in the study.

Table S3. Capacity based on anodic peak area of CV measurement.

	Capacity / mAh/g		
**Scan number**	**AAPSi**	**THCPSi**	**TCPSi**
**1**	2600	1500	1600
**2**	3400	2300	1500
**3**	3500	2500	1900
**4**	3500	2400	1900
**5**	3400	2200	1800
**6**	3400	2200	1800
**7**	3300	2100	1800
**8**	3100	2000	1700
**9**	3100	1900	1600
**10**	2800	1900	1600

Table S4. External surface area of PSi particles based on the particle size measurements using Mie theory and assumption of spherical particles.

Sample	External surface area of PSi particles (m^2/g)
TCPSi 2:	7.7
TCPSi 7:	2.0
TCPSi 12:	0.9
TCPSi 24:	0.3
TCPSi 38:	0.2

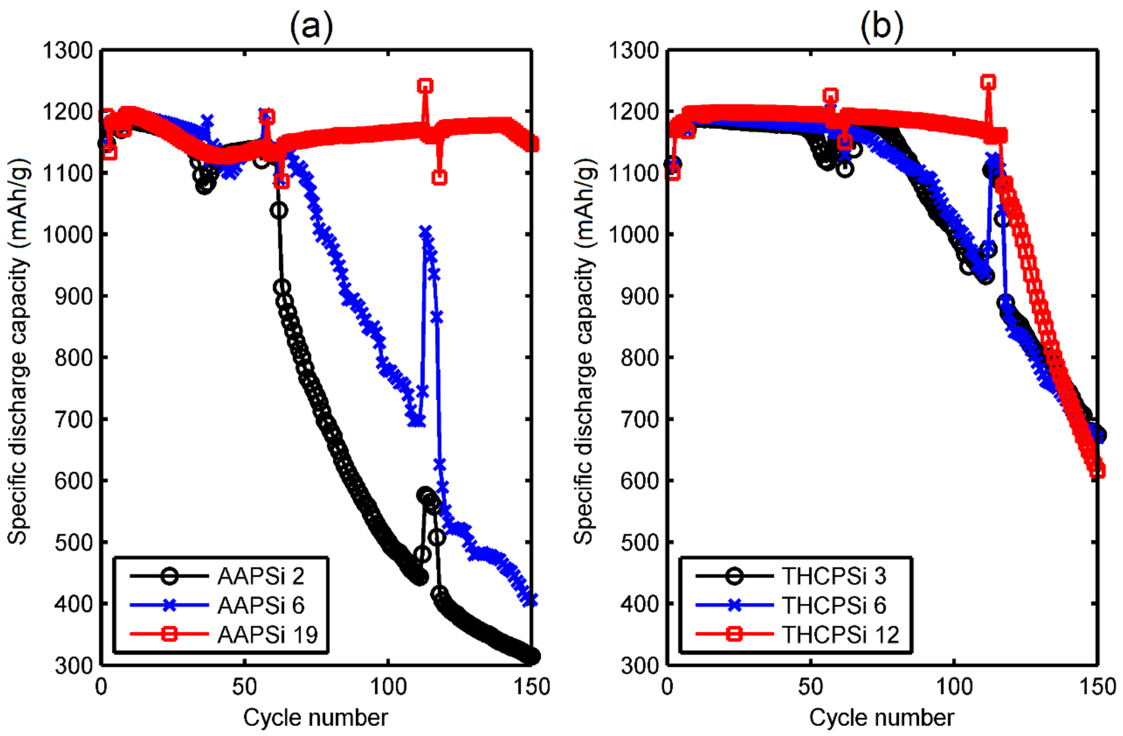

Figure S2. Galvanostatic capacity retention results for **(a)** the AAPSi and **(b)** the THCPSi samples. Cycling was done periodically: 5 x 0.1C + 50 x 0.2C. 1C equals to 4200 mA/g. Average mass loading of silicon is 0.8 mg/cm^2 for both types of samples.

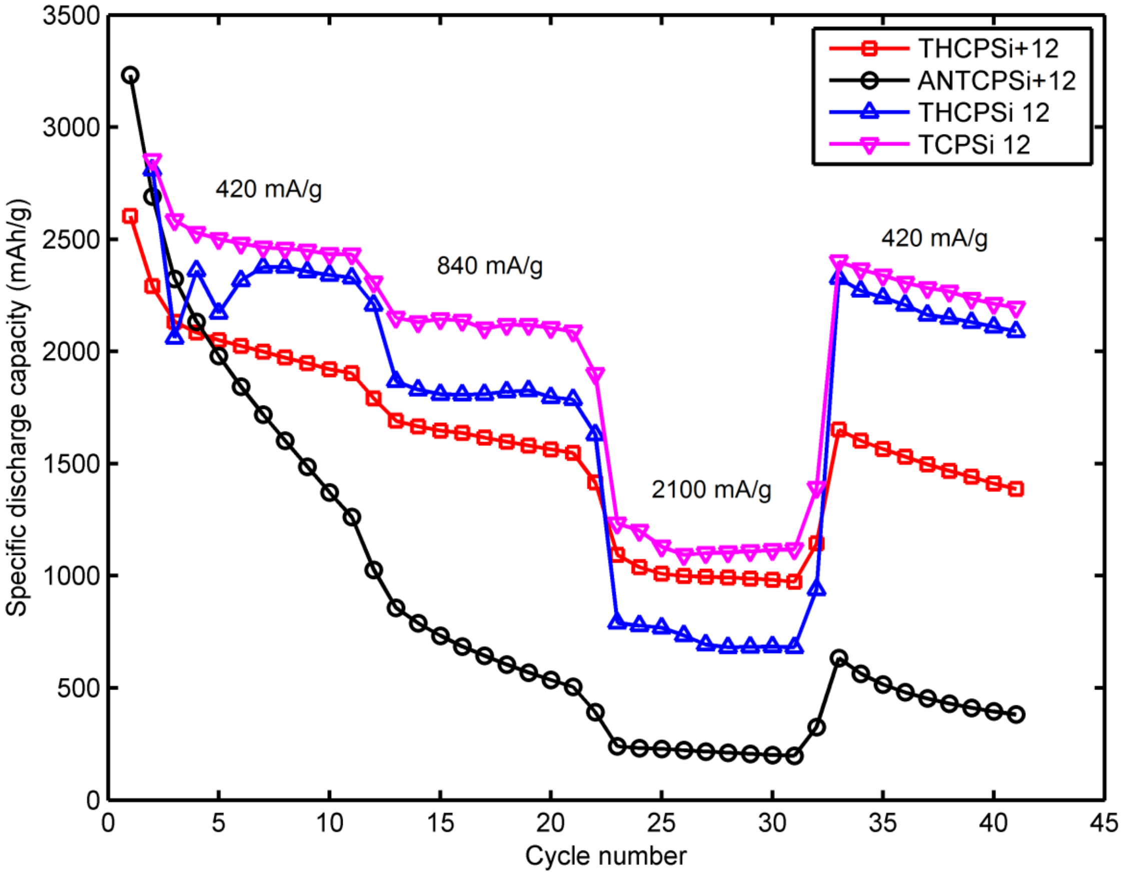

Figure S3. Galvanostatic rate capability results for lightly doped PSi together with comparable results for highly doped PSi. Mass loading of silicon is 0.9 mg/cm^2.

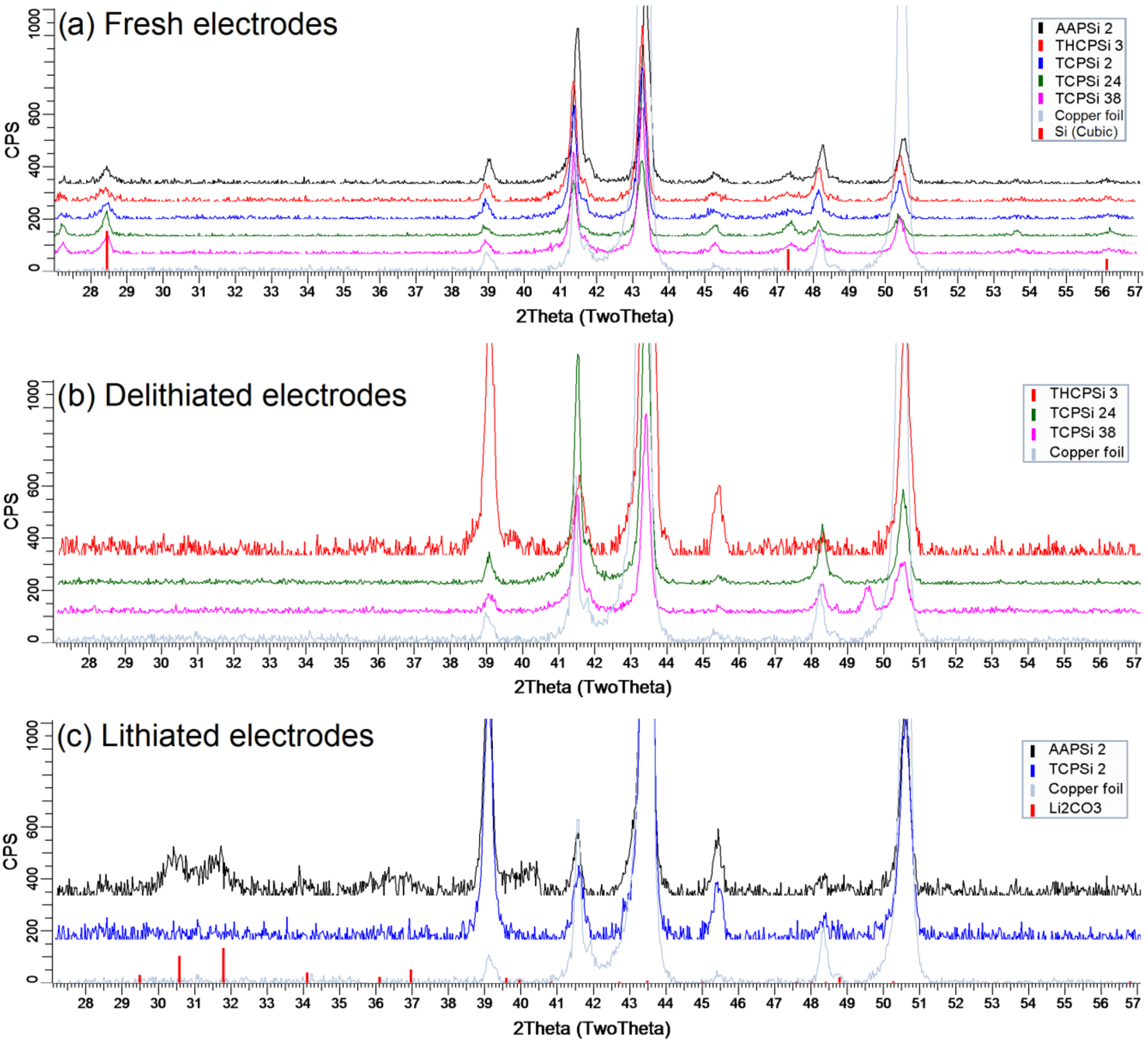

Figure S4. Diffractograms for **(a)** fresh electrodes, **(b)** delithiated electrodes after 42 cycles and **(c)** lithiated electrodes after 42 cycles. The red dashes correspond to (a) characteristic silicon peaks or to (c) lithium carbonate.

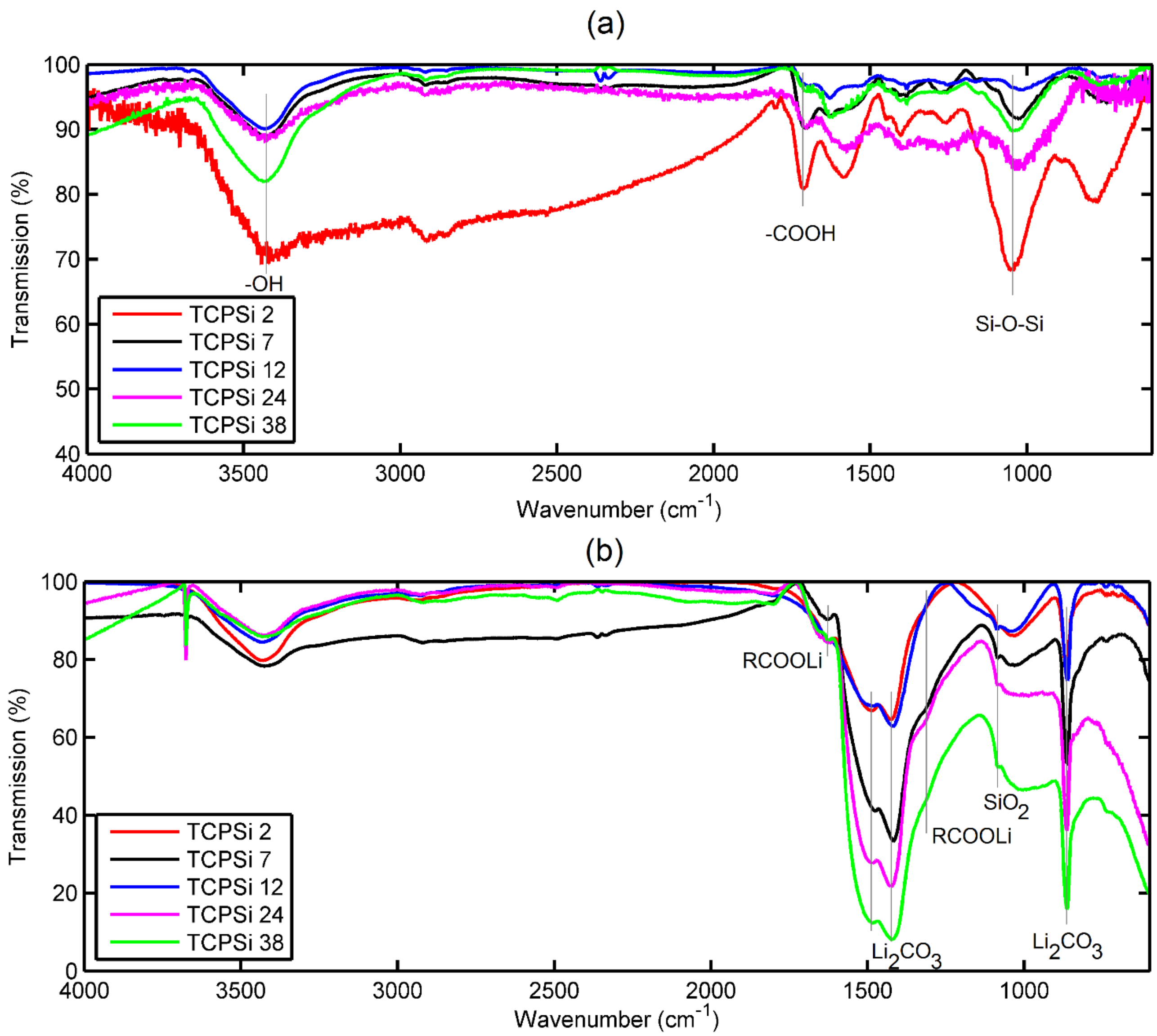

Figure S5. FTIR spectra of **(a)** fresh electrodes and of **(b)** cycled electrodes from the TCPSi samples.

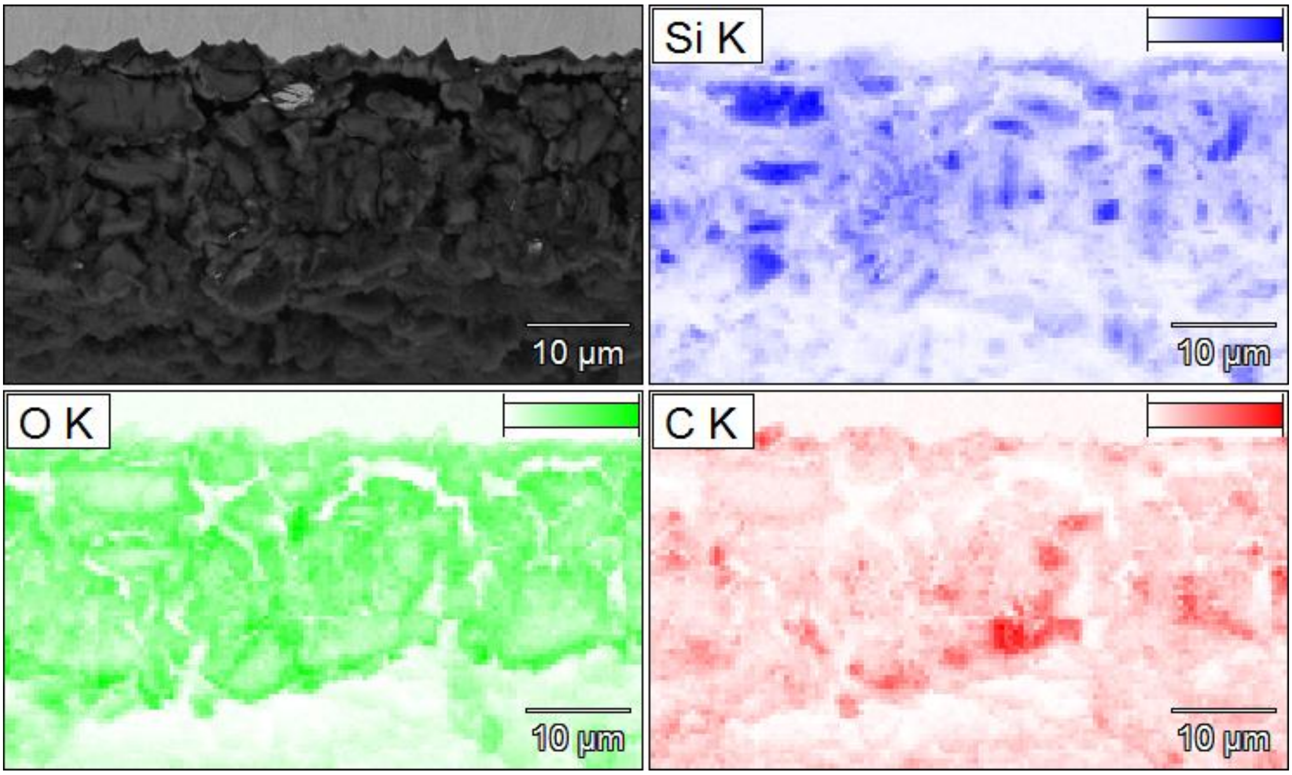

Figure S6. SEM image and corresponding EDS mapping from ion cut TCPSi 38 electrode after 300 cycles. Silicon (Si), oxygen (O) and carbon (C) mapping after 300 cycles. The top part in each figure is the copper current collector.

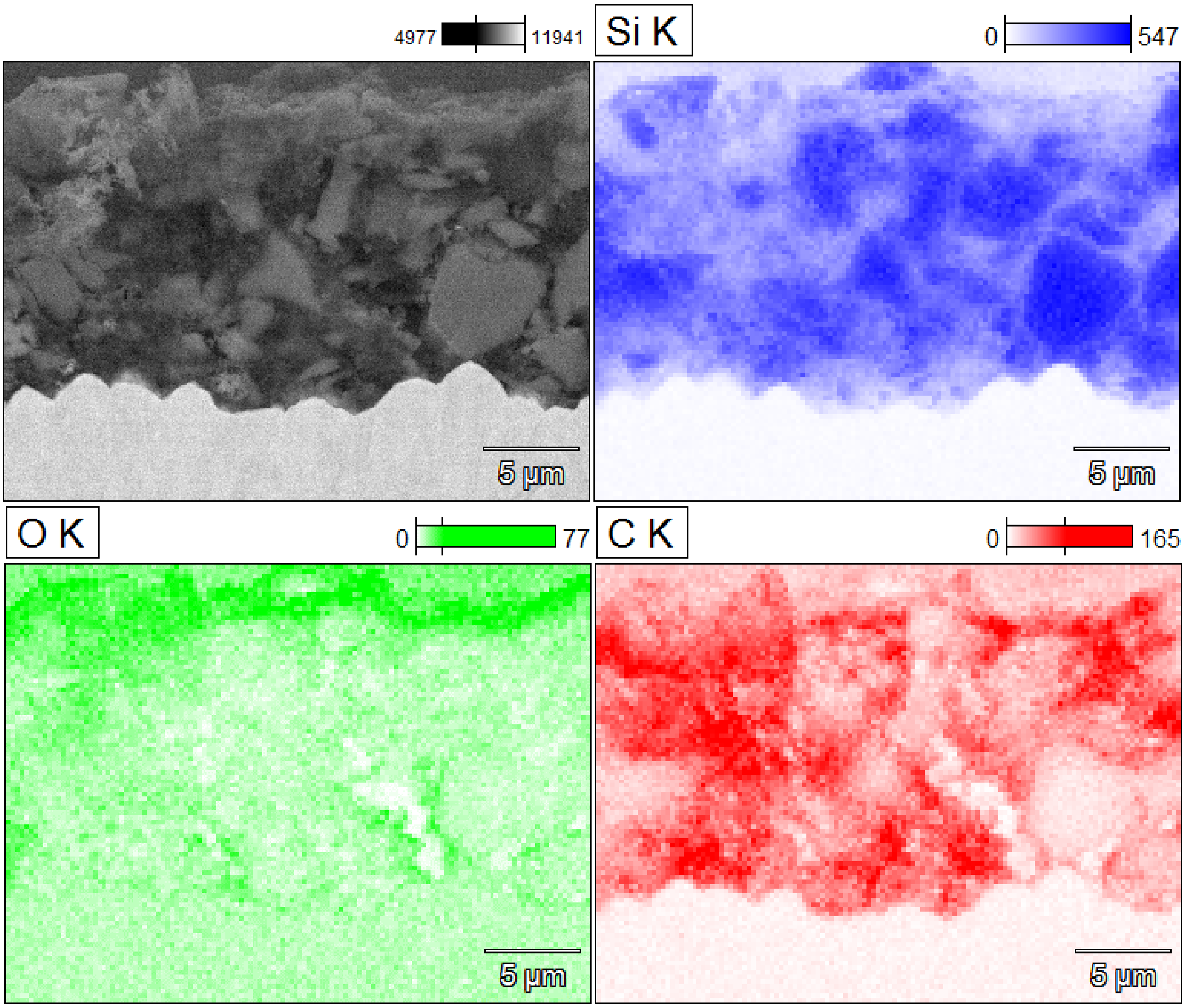

Figure S7. EDS results from the fresh ion cut electrode (THCPSi) with silicon (Si), oxygen (O) and carbon (C) mapping. The bottom part in each figure is the copper current collector.

References

1. Lehto., V.-P., Vähä-Heikkilä, K., Paski, J., Salonen, J., Use of thermoanalytical methods in quantification of drug load in mesoporous silicon microparticles, *Journal of Thermal Analysis and Calorimetry* **80**, 393-397 (2005).
